# 

**Published:** 1852-08

**Authors:** 


SUBSCRIBERS IN ARREARS

Will please remit to the Publishers at their risk the amounts severally due by them, obtaining from the Post Master a certificate of the amount enclosed. Messrs. C. W. James, Israel E. James, Henry M. Lewis, and their Assistants, are the only persons authorised to collect money on account of this Journal.

LINDSAY & BLAKISTON, *Publishers*,
Philadelphia.

